# Factors associated with male involvement in the prevention of mother to child transmission of HIV, Midlands Province, Zimbabwe, 2015 - a case control study

**DOI:** 10.1186/s12889-016-2939-7

**Published:** 2016-04-14

**Authors:** Annamercy Makoni, Milton Chemhuru, Cleopas Chimbetete, Notion Gombe, More Mungati, Donewell Bangure, Mufuta Tshimanga

**Affiliations:** Department of Community Medicine, University of Zimbabwe, Harare, Zimbabwe; Ministry of Health and Child Care, Harare, Zimbabwe

**Keywords:** PMTCT, Male involvement, Midlands Province, Zimbabwe

## Abstract

**Background:**

Uptake of and adherence to the prevention of mother to child transmission of HIV (PMTCT) interventions are a challenge to most women if there is no male partner involvement. Organizations which include the National AIDS Council and the Zimbabwe AIDS Prevention Project- University of Zimbabwe have been working towards mobilizing men for couple HIV testing and counseling (HTC) in antenatal care (ANC). In 2013, Midlands province had 19 % males who were tested together with their partners in ANC, an increase by 9 % from 2011. However, this improvement was still far below the national target, hence this study was conducted to determine the associated factors.

**Methods:**

A1:1 unmatched case control study was conducted. A case was a man who did not receive HIV testing and counseling together with his pregnant wife in ANC in Midlands province from January to June 2015. A control was a man who received HIV testing and counseling together with his pregnant wife in ANC in Midlands province from January to June 2015. Simple random sampling was used to select 112 cases and 112 controls. Epi Info statistical software was used to analyze data. Written informed consent was obtained from each study participant.

**Results:**

Independent factors that predicted male involvement in PMTCT were: having been previously tested as a couple (aOR) 0.22, 95 % CI = 0.12, 0.41) and having time to visit the clinic (aOR) 0.41, 95 % CI = 0.21, 0.80). Being afraid of knowing one’s HIV status (aOR 2.22, 95 % CI = 1.04, 4.76) was independently associated with low male involvement in PMTCT.

**Conclusion:**

Multiple factors were found to be associated with male involvement in PMTCT. Routine PMTCT educational campaigns in places where men gather, community based couple HTC and accommodating the working class during weekends are essential in fostering male involvement in PMTCT thereby reducing HIV transmission to the baby.

## Background

The prevention of mother to child transmission of the immunodeficiency virus (HIV) (PMTCT) program offers an opportunity to capture pregnant women with their partners in order to prevent the transmission of HIV to the baby [1]. Transmission of HIV from the mother to the baby occurs during pregnancy, labor and breastfeeding [1]. Testing as a couple in antenatal care (ANC) promotes couple communication, mutual disclosure, mutual decision making on issues to do with safer sex, infant feeding and family planning. Support from a male partner makes a woman adhere to PMTCT [1]. Male partner involvement in PMTCT may reduce the risk of mother to child transmission of HIV (MTCT) by more than 40 % [2]. Some women who test negative during ANC still seroconvert during pregnancy and post-delivery hence the importance of couple HTC.

The PMTCT program in Zimbabwe follows guidelines by the World Health Organization (WHO) [3]. The first pillar aims to keep couples HIV negative. This can be achieved through provision of voluntary counseling and testing (VCT) services, early diagnosis and treatment of sexually transmitted infections (STIs) and condom programming. The second pillar aims to prevent unintended pregnancies among the HIV positive women. This is achieved by provision of VCT services and effective family planning. The third pillar aims to prevent HIV transmission from mothers to their infants, during pregnancy, labor, delivery and breastfeeding. It is achieved through provision of ART, safe delivery practices and safer infant feeding practices. The fourth pillar aims to provide treatment, care and support to the infected and affected. This is achieved by provision of lifelong ART, psychosocial support and early infant diagnosis of HIV and treatment [3].

It was estimated that 35.3 (32.2–38.8) million people were living with HIV globally in 2012 with 2.3 million new HIV infections, a 33 % decline from 3.4 million in 2001 [4]. Worldwide, 600 000 children are newly infected by HIV annually [5]. Of the new HIV pediatric infections, 90 % result from the preventable MTCT [5]. Over 900 000 pregnant women living with HIV accessed PMTCT services globally, a coverage of 62 % in 2012 [6].

Sub Saharan Africa reported 65 % PMTCT coverage in 2012 [6]. An estimated 260 000 children became infected of which more than 90 % contracted HIV from their mothers [6]. Research has highlighted a number of barriers to achieving comprehensive PMTCT coverage in Sub Saharan Africa. In many African countries offering PMTCT, men are the decision makers. The majority of women need the consent of their partners to accept HIV testing and PMTCT.

Zimbabwe’s HIV burden remains significantly high with an estimated 1.4 million adults and children living with HIV in 2012 [7]. About 15 000 new pediatric HIV infections are occurring annually and 90 % of them are through MTCT [3]. In 2013, 95 % of HIV positive women who booked for ANC received antiretroviral therapy (ART) for PMTCT [7]. As implementation moves towards lifelong ART for pregnant and lactating women, engagement of male partners in the PMTCT program is crucial. Male partner HIV testing in ANC continues to be a challenge in the country [7].

All the ten provinces in Zimbabwe reported a low proportion of male partners who got tested for HIV in ANC [8]. Between January and March 2014, the proportion of pregnant women tested with their male partners in ANC was highest in Mashonaland East and lowest in Harare, 31 % and 7 % respectively. In Midlands province 22 % of the women were tested with their male partners during the same period [9].

Community health workers (CHWs) assist by mobilizing men to accompany their pregnant wives for PMTCT as part of the comprehensive package of ANC. Organizations like the National AIDS Council (NAC) and the Zimbabwe AIDS Prevention Project- University of Zimbabwe (ZAPP-UZ) have been working towards mobilizing men for couple HU and counseling in ANC. Midlands province recorded a rise in the proportion of male partners tested for HIV in ANC from 10 % (*n* = 4 654) in 2011 to 19 % in 2013 [3, 8]. This improvement in male partner involvement was still below the national target of 30 % by 2015. Majority of districts in the province are far below the national target. In 2013, the highest, medium and lowest proportions of male partners tested for HIV with their wives in ANC were 21 % (*n* = 517) in Zvishavane, 16 % (*n* = 561) in Gweru, 11 % in Kwekwe (*n* = 489) and 10 % (*n* = 192) in Chirumhanzu districts respectively. HIV testing of male partners in ANC is a proxy indicator used to assess involvement of male partners in PMTCT in Zimbabwe. The study therefore seeks to determine factors associated with male involvement in PMTCT in Midlands Province focusing on couple HIV testing and counseling in ANC.

## Methods

A 1:1 unmatched case control study was conducted for primary participants (male partners) to assess the factors associated with male involvement in PMTCT. A case control study design was chosen because of its ability to allow for the evaluation of a wide range of factors for a single outcome. A case control study design is efficient in terms of both time and costs, relative to other analytic approaches.

### Study population

Male partners of pregnant women attending ANC or of women who delivered during the period January to June 2015 in Zvishavane, Gweru and Kwekwe districts of Midlands province. A case was a man who did not receive HIV testing and counseling together with his pregnant wife in ANC in Midlands province from January to June 2015. A control was a man who received HIV testing and counseling together with his pregnant wife in ANC in Midlands province from January to June 2015.

### Study setting

The study was conducted in Midlands province in the high, medium and low performing districts with both rural and urban settings, that is, Zvishavane, Gweru and Kwekwe districts respectively in Midlands province, Zimbabwe. The decision to select 3 district hospitals with rural and urban settings was guided by the assumption that information generated from the three districts was representative of the whole province. Male partners who were staying together with their wives during pregnancy in Midlands province and willing to participate were included in the study.

### Permission to proceed and ethical considerations

Permission to conduct the study was obtained from the Provincial Medical Director (PMD), Midlands province, Gweru, Kwekwe and Zvishavane District Medical Officers (DMOs) and the Health Studies Office (HSO). Approval and permission to proceed with the study was obtained from the Medical Research Council of Zimbabwe (MRCZ), approval number: MRCZ/B/878.

A written informed consent was obtained from each study participant. The aim of the study was explained and the participants were informed that they were free to withdraw at any time during the interview. Confidentiality was assured by informing the participants that the results of the study would be used for study purposes only. No incentives were given to study participants for participating or as a way to motivate them to participate. Ethical approval was obtained from the Joint Research Ethics Committee (JREC), approval number: JREC/130/15.

### Primary participants

Sample size was calculated using Stat Calc and based on a study by Dyodo et al. “Factors associated with male involvement in maternal health care services in Uganda.” Assuming 80 % power at 95 % CI, a precision of 5 %, and a case control of 1:1 with a prevalence of 46 % of employed men accompanying their partners for couple HTC in ANC, Odds Ratio (OR) =2.22, the minimum sample size was 112 cases and 112 controls with 10 % attrition.

### Controls

Men who accompanied their partners and got tested for HIV as a couple in ANC were randomly recruited for the study. Each male partner’s name in the HTC register was allocated a number. Microsoft Excel software was used to randomly select names from the list. The math and trigonometry function RANDBETWEEN was used. The picked names were the cases that were then followed up for interviews. Random numbers were generated until the required sample size was reached.

### Cases

Names of women who were tested alone in ANC were randomly selected from the ANC register using the computer-generated random numbers. Each woman’s name in the ANC register was allocated a number. Microsoft Excel software was used to randomly select names from the list. The math and trigonometry function RANDBETWEEN was used. Contact details of the selected names were used to follow up the male partners for interviews. Random numbers were generated until the required sample size was reached.

### Data collection

An interviewer administered questionnaire was used to collect data from cases and controls on socio-demographic characteristics, health service related factors, socio-cultural factors and knowledge on PMTCT. Both cases and controls were interviewed in their communities. Men who received HTC with their partners were randomly selected from the HTC register and followed up for interviews in the community. Women who were tested alone in ANC were randomly selected from the ANC register. Contact details of these women were used to follow up their male partners for interviews. Appointments were made with male partners who were busy, not available or not ready for an interview, to interview them at their workplace, at home after work or during the weekend.

### Questionnaire validation procedures

The questionnaire was circulated among twenty program managers in the province for review. Feedback was received and corrections were made accordingly. It was then edited as follows: order of questions for coherency, rephrasing of some questions for clarity, more questions were added to the questionnaire in order to address all the objectives adequately. Pretesting of the questionnaire was done in Shurugwi district which was not part of the study, to assess for clarity of questions, validity of the questionnaire, average time needed to complete a questionnaire, availability of study participants for interviews, sensitive questions were also noted which were to be asked indirectly. Data were collected from 10 % of the study sample size, 11 cases and 11 controls. A mini study analysis was conducted and the following editions were done: Questions which were not clear to interviewees were rephrased and some questions which were a repetition of what was previously asked were removed. After all these procedures, the questionnaire was deemed valid and consistent with the conceptual framework and objectives.

### Data analysis

The questionnaire was created in Epi info™ for data analysis. Epi Info™ (3.5.4) was used to: generate frequencies, calculate odds ratios (ORs), *p*-values and 95 % Confidence Interval (CI). Forward stepwise logistic regression analysis was done to determine the independent factors associated with male involvement in PMTCT. Variables were introduced one by one, starting with the most significant. All variables that were associated with male involvement in PMTCT with a *p*-value ≤ 0.25 were included in the logistic regression model.

## Results

### Descriptive epidemiology

Table 1 illustrates socio-demographic characteristics of primary study participants. Study participants were comparable with respect to age, partner’s age, level of education, partner’s level of education, employment status, partner’s employment status, average family monthly income and place of residence.

**Table 1:** Socio-demographic characteristics of cases and controls, Midlands Province, Zimbabwe, 2015

Variable	Category	Cases	Controls	*p* value
		*n* = 112 (%)	*n* = 112 (%)	
Age	21-30 31–40 41–50 >50	47 (42.0) 53 (47.3) 11 (9.8) 1 (0.9)	48 (42.9) 49 (43.7) 14 (12.5) 1 (0.9)	0.54
Median age (years)		32 (Q_1_ = 27; Q_3_ = 36)	32 (Q_1_ = 27; Q_3_ = 37)	
Partner’s Age	<21 21–30 31–40 41-50	17 (15.2) 63 (56.2) 31 (27.7) 1 (0.9)	15 (13.4) 69 (61.6) 24 (21.4) 4 (3.6)	0.54
Partner’s median age (yrs)		25 (Q_1_ = 22;Q_3_ = 31)	25 (Q_1_ = 22;Q_3_ = 30)	
No. of children	0-1 2–3 >3	36 (32.1) 57 (50.9) 19 (17.0)	46 (41.1) 56 (50) 10 (8.9)	0.17
Place of residence	Rural Urban	23 (20.5) 89 (79.5)	22 (19.6) 90 (80.4)	0.87
Level of education	Primary Secondary Tertiary	6 (5.3) 99 (88.4) 7 (6.3)	4 (3.6) 98 (87.5) 10 (8.9)	0.52
Partner’s level of education	Primary Secondary Tertiary	10 (8.9) 97 (86.6) 5 (4.5)	8 (7.1) 96 (85.8) 8 (7.1)	0.62
Religion	None Traditional Orthodox Pentecostal Islam Apostolic	32 (28.6) 1 (0.9) 17 (15.1) 30 (26.8) 1 (0.9) 31(27.7)	21 (18.7) 1 (0.9) 29 (25.9) 34 (30.4) 1 (0.9) 26 (23.2)	0.09
Employment Status	Formal Informal Unemployed	45 (40.2) 49 (43.8) 18 (16.0)	55 (49.1) 37 (33.0) 20 (17.9)	0.72

### Analytic epidemiology

Table 2 shows the socio-cultural factors associated with male involvement in PMTCT. Men who could get time to visit the clinic with their wives (OR 0.31, 95 % CI = 0.17, 0.56), received feedback from their partners on ANC services (OR 0.32, 95 % CI = 0.15, 0.71), were previously tested for HIV with their wives as a couple (OR 0.22, 95 % CI = 0.12, 0.38) and those who were comfortable receiving HTC at a local clinic (OR 0.38, 95 % CI = 0.18, 0.79) were significantly more likely to be involved in PMTCT. Men who reported to be afraid of knowing their HIV status (OR 2.37, 95 % CI = 1.19, 4.70) were significantly less likely to be involved in PMTCT.

**Table 2:** Socio-cultural factors associated with male involvement in PMTCT, Midlands Province, Zimbabwe, 2015

Factor		Cases	Controls	OR	95 % CI
		*n* = 112 (%)	*n* = 112 (%)		
Previously tested as a couple	Yes No	28 (25.0) 84 (75.0)	68 (60.7) 44 (39.3)	0.22^**^	0.12, 0.38
Have time to visit clinic	Yes No	64 (57.1) 48 (42.9)	91 (81.3) 21 (18.7)	0.31^**^	0.17, 0.56
Received feedback from wife on ANC services	Yes No	86 (76.8) 26 (23.2)	102 (91.1) 10 (8.9)	0.32^**^	0.15, 0.71
Comfortable receiving HTC at local clinic	Yes No	85 (75.9) 27 (24.1)	100 (89.3) 12 (10.7)	0.38^*^	0.18, 0.79
Afraid of knowing HIV status	Yes No	30 (26.8) 82 (73.2)	15 (13.4) 97 (86.6)	2.37^*^	1.19, 4.70
Drinks alcohol	Yes No	57 (50.9) 55 (49.1)	44 (39.3) 68 (60.7)	1.92	0.90, 4.13
Have >1 sexual partner	Yes No	30 (26.8) 82 (73.2)	20 (17.9) 92 (82.1)	1.50	0.80, 2.80

Level of statistical significance: *p < 0.05

Table 3 summarizes the analysis of health service related factors associated with male involvement in PMTCT. Men who perceived health workers to be welcoming (OR 0.40; 95 % CI = 0.18, 0.89), clinic’s operating time to be convenient (OR 0.46; 95 % CI = 0.23, 0.93) and who had ever heard about PMTCT (OR 0.45; 95 % CI = 0.23, 0.86) were significantly more likely to be involved in PMTCT. Men who perceived that there were delays in ANC service provision were significantly less likely to be involved in PMTCT (OR 1.94; 95 % CI = 1.12, 2.52).

**Table 3:** Health service related factors associated with male involvement in PMTCT, Midlands Province, Zimbabwe, 2015

Factor		Cases	Controls	OR	95 % CI
		*n* = 112 (%)	*n* = 112 (%)		
Clinic operating time convenient	Yes No	97 (86.6) 15 (13.3)	84 (75.0) 28 (25)	0.46^*^	0.23, 0.93
Ever heard about PMTCT	Yes No	80 (71.4) 32 (28.6)	95 (84.8) 17 (15.2)	0.45^*^	0.23, 0.86
Perceived clinic staff welcoming	Yes No	102 (91.1) 10 (8.9)	90 (80.4) 22 (19.6)	0.40^*^	0.18, 0.89
Perceived delays at the clinic	Yes No	32 (28.6) 80 (71.4)	49 (43.8) 63 (56.2)	1.94^*^	1.12, 3.39
Perceived confidentiality at clinic	Yes No	110 (98.2) 2 (1.8)	105 (93.7) 7 (6.3)	0.27	0.06, 1.35
Perceived couple HTC as important	Yes No	108 (96.4) 4 (3.6)	101 (90.2) 11 (9.8)	0.34	0.10, 1.10
Health facility accessible	Yes No	107 (95.5) 5 (4.5)	101 (90.2) 11 (9.8)	0.43	0.14, 1.28
Lives within ≤5 km from the clinic	Yes No	90 (80.4) 22 (19.6)	83 (74.1) 29 (25.9)	0.70	0.37, 1.31
Invited to the clinic by wife	Yes No	60 (54.1) 52 (45.9)	71 (63.4) 41 (36.6)	1.47	0.86, 2.52

Level of statistical significance: *p < 0.05, **p < 0.01

Table 4 shows the independent factors found to be associated with male involvement in PMTCT. Having been previously tested as a couple (aOR 0.22, 95 % CI = 0.12, 0.41) and having time to visit the clinic with wife (aOR 0.41, 95 % CI = 0.21, 0.80) were the independent factors associated with male partners being more likely to be involved in PMTCT. Being afraid of knowing one’s HIV status (aOR 2.22 95 % CI = 1.04, 4.76) was an independent factor associated with male partners less likely to be involved in PMTCT.

**Table 4:** Independent factors associated with male involvement in PMTCT, Midlands Province, Zimbabwe, 2015

Factor	Crude OR	Adjusted OR	*p*-value
	(95 % CI)	(95 % CI)	
Previously tested as a couple	0.22 (0.12, 0.38)	0.22 (0.12, 0.41)	<0.01
Have time to visit clinic	0.31 (0.17, 0.56)	0.41 (0.21, 0.80)	0.01
Afraid of knowing HIV status	2.37 (1.19, 4.70)	2.22 (1.04, 4.76)	0.04

**Fig. 1:**
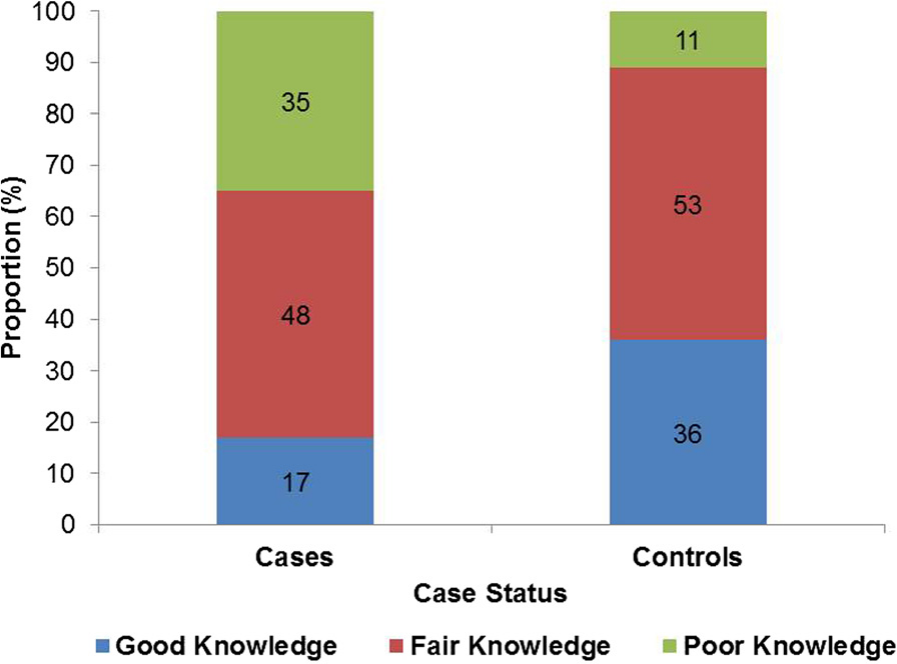
Knowledge of Male Partners on PMTCT, Midlands Province, 2015

Knowledge was assessed on a 5 point Likert Scale where a scale of 1–2 was poor knowledge, 3 was fair knowledge and 4–5 was good knowledge. Figure 1 shows knowledge levels of male partners on PMTCT. Majority of cases and controls had fair knowledge on PMTCT, 48 % and 53 % respectively. However, a small proportion of cases, 17 % had good knowledge as compared to 36 % of controls. More cases had poor knowledge on PMTCT, 35 % as compared to 11 % of controls.

## Discussion

A 1:1 unmatched case control study design was used to determine factors associated with male partner involvement in PMTCT. Socio-cultural (history of couple HTC and fear of knowing HIV status), health service related factors (staff friendly and welcoming, ever heard about PMTCT) and knowledge) were found to be associated with male involvement in PMTCT.

Previous couple HIV testing and counseling was associated with male involvement in PMTCT. Couples who are aware of their status are not afraid of HIV testing and counselling unlike those who are not aware. This implies that promoting couple HIV testing and counselling in all other HTC entry points might enhance male involvement in PMTCT. Similar findings were reported by Morfaw et al. in sub Saharan Africa in 2013 [10]. Being afraid of knowing HIV status was associated with male partners less likely to be involvement in PMTCT.

Men who had more than one sexual partner were less likely to be involved in PMTCT. In a study by Ditekemena et al. in Kinshasa, it was found out that men with one sexual partner were twice more likely to be involved in PMTCT [11]. Similar findings were reported by Kang’oma et al. in Malawi where they found that fear of HIV testing among men who engaged in extra marital affairs was the main reason why men did not participate in PMTCT services [12]. Men who engage in multiple unprotected sexual relationships are at a higher risk of acquiring HIV. About 45 % (*n* = 100) of the male partners interviewed in this study reported that they engaged in extra marital affairs. Majority of these men refused to accompany their wives for PMTCT due to the fear of knowing their HIV status which might result in stigma, discrimination, domestic violence or abandonment by wife if positive. From the qualitative data, it was also highlighted by three quarters of the respondents that the fear of HIV test results was the main barrier to male involvement in PMTCT. Men who perceive themselves at risk of HIV refuse to go for couple HTC. This implies the need for more HIV and AIDS educational and behaviour change communication programs for male partners in order to address issues to do with culture and the benefits of knowing one’s HIV status.

Having time to accompany wife for PMTCT was associated with male involvement in PMTCT. Male partners who could get time were more likely to accompany their wives than those who were always busy with other commitments. Men are usually limited by shortage of time due to various commitments mainly in urban settings. Eighty three percent of the respondents were either formally or informally employed. However it is still possible for a male partner to take a day off considering that in Zimbabwe, workers in the formal sector get twelve days annual leave and a number of other leave days. Men in the informal sector need to plan their time so that they can accompany their wives for PMTCT.

Similar findings were reported in a systematic review by Morfaw et al. in sub Saharan Africa in 2013 where lack of time and the non-invitations to the health facility were the main reasons for low male participation [10]. It is interesting to note that in this study being employed was associated with the likelihood of male partners being involved in PMTCT. This was contrary with other studies were the association was found to be significant [13, 14].

Men who reported to be comfortable being tested at their local clinic were more likely to be involved in PMTCT. HIV testing at the local clinic may be associated with stigma and discrimination thereby hindering male partners from accompanying their wives for PMTCT. Men would prefer to be tested elsewhere outside their nearest health facility. Similar findings were reported by Ditekemena et al. in a randomized control study which was conducted in Kinshasa, DRC. It was found that the majority of male partners preferred to be tested for HIV in non-health service settings like churches [11]. This implies that sensitizing and referring men for PMTCT in all other entry points might improve their involvement. Similar findings were reported by Ncube et al. in Bulawayo, Zimbabwe, where they found out that being comfortable testing at the clinic where partner goes for ANC was one of the predictors of male participation in PMTCT [15].

Male partners who received feedback from their wives on ANC issues were significantly more likely to be involved in PMTCT than those who did not receive any feedback. Giving feedback to a partner might imply good couple communication and hence acceptance by the male partner to be involved in PMTCT despite other limiting factors. In other studies barriers to male involvement in PMTCT programs included lack of couple communication and the unwillingness of women to have their male partners involved because they feared domestic violence, stigmatization and divorce if HIV positive. Other studies reported poor communication on ANC services and the importance of couple HTC between partners was associated with poor male involvement in PMTCT [10, 16].

Having ever heard about PMTCT was significantly associated with male involvement in PMTCT. However, the association was overestimated by the fear of knowing one’s HIV status. Male partners who heard about PMTCT appreciated the importance of their involvement in the program and were more likely to participate. In this study it was found out that men who had never heard about PMTCT did not appreciate it as an important service, hence did not participate. The main sources of information highlighted by the respondents were the clinic/health worker, family/friend and the radio and television. Nsagha et al. also reported the clinic/health worker and television as the main sources of PMTCT information in Cameroon [17].

The friendliness and welcoming of male partners by health workers was significantly associated with male involvement in PMTCT. Men need to feel that they are important and are part of the pregnancy when they accompany their wives for ANC. In a descriptive qualitative study conducted by Adelekam et al. in Nigeria, married men agreed that it was important to accompany their wives for ANC. However, they highlighted that before accompanying their wives, they must feel needed [18]. Similar findings by Byamugisha et al. in Eastern Uganda, who reported that harsh treatment of men by health care providers, discouraged them from being involved in PMTCT [16].

In Uganda, it was found that the unwelcoming attitude of health workers and the fear of being harassed by health workers were some of the reasons contributed to low male involvement [14].

Men who reported that the clinic’s operating time was convenient were more likely to be involved in PMTCT that those who reported that the time was inconvenient. Most men whether formally employed, informally employed or unemployed run around working for the family during the week and would be found at home during the weekends. Men who work at least five days a week and cannot get off days from work find the clinic’s operating time inconvenient for them. This implies that if clinics would open during weekends, more men would be available to accompany their wives for PMTCT. If men are given formal invitation letters by the clinic, they can present them at their workplaces and ask for a day off.

Men who perceived delays in service provision at the health facility were significantly less likely to be involved in PMTCT. There were a number of activities that took place in ANC in Midlands province as highlighted by key informants and these included, group health education, booking, examination, weighing, paying and HTC. These activities might take an average of two hours. In a study by Byamugisha et al. in Eastern Uganda in 2010, it was found that ANC service providers take time to deliver services to clients, hence male partners are not in a position to spend more time at ANC clinics [16]. Short waiting time of less than 30 min was found to increase male involvement in maternal health services in Uganda [14]. This implies that attending couples first and increasing the number of nurses in ANC to avoid delays might improve male partner involvement in PMTCT. Giving first preference to those who bring their male partners is also very important in that it encourages other women to convince their partners to accompany them.

Men who perceived couple HTC for PMTCT as important were more likely to be involved in PMTCT but was not statistically significant in this study. Ncube et al. in Bulawayo City, Zimbabwe, found out that having the attitude that it was important for couples to get counseled and tested for PMTCT was significantly associated with male participation in PMTCT [15]. The same study also found out that men who believed that it was important for them to attend HIV testing and counseling together with their female partners were more likely to participate in PMTCT [15]. Madzima et al. found out in Zvimba district that male partners who knew the benefits of PMTCT were more likely to be involved in PMTCT [19]. This implies that routine advocacy and social mobilization activities on the importance and benefits of male involvement in PMTCT are necessary to improve male involvement.

In this study men who were invited for couple HTC in ANC were less likely to be involved in PMTCT though not statistically significant. Men might not value verbal invitations by their wives, an initiative which was being used by all the clinics, hence the need for formal invitation letters. In a randomized control trial conducted in Zvimba district, Zimbabwe in 2010 by Madzima et al., it was found that male partners invited by a formal letter were more likely to participate in PMTCT [19]. This implies that male partner involvement may improve if the clinics write formal invitation letters to male partners explaining the importance of their involvement. In a study by Dyogo et al. in Uganda, male partners who were invited through formal letters were significantly more likely to be involved in maternal health services [14].

Knowledge levels were significantly high among men who were involved in PMTCT. This might be due to the exposure to PMTCT information at the health facilities. Men who are knowledgeable about PMTCT get involved and support their wives because they understand the benefits. In this study, men who supported their wives joining the PMTCT program were more likely to be involved in PMTCT but not statistically significant. High knowledge levels enhance program acceptability an uptake. Similar findings were reported in a study by Ncube et al. in Bulawayo City where the majority of men who participated in PMTCT had good knowledge on PMTCT whilst the majority who did not participate in PMTCT had poor knowledge on PMTCT [15]. In a study by Tshibumbu et al. in Zambia, knowledge of PMTCT was the strongest factor which was positively associated with male partner involvement in PMTCT [20]. Similar findings were also reported in other studies [10, 19, 21, 22]. This implies that exposing men to adequate information on the importance of their involvement in PMTCT might improve their involvement. Dyogo et al. in Uganda found out from the qualitative data that lack of knowledge on the benefits and need of male involvement in maternal health care services was reported to contribute to low male involvement [14].

### Limitation of the Study

There was a possibility of recall bias whereby both cases and controls could have forgotten about history of HIV testing and counseling as a couple. This was minimized by confirming with their wives whether they were once tested as a couple before.

There was a possibility that men who were to accompany their wives later were likely to be recruited as cases during the time the study was conducted. This was avoided by recruiting men whose wives had attended the forth, which is the last ANC visit and those who had just delivered.

Social desirability bias cannot be excluded from this study whereby cases and controls could have responded in a way to please the researcher. It can take the form of over-reporting good behavior or under-reporting bad or undesirable behavior. This bias interferes with the interpretation of average tendencies as well as individual differences. Indirect questioning was used to reduce such bias.

## Conclusions

Multiple factors were found to be associated with male involvement in PMTCT in Midlands province. Prior couple HIV testing and counseling and having time to visit the clinic with wife were the independent factors associated with male partners being more likely to be involved in PMTCT. Being afraid of knowing one’s HIV status was an independent factor associated with male partners less likely to be involved in PMTCT. Knowledge levels were significantly higher among men who were involved in PMTCT.

### Recommendations

This study recommends the implementation of innovative strategies to reach out to all men with partners in the reproductive age group. These include PMTCT educational campaigns during weekends in beer halls, churches and the community, involvement of stakeholders (local chiefs, religious leaders, political leaders, private hospitals, New Start Centers and Non- Governmental Organizations) in PMTCT advocacy and social mobilization and community based couple HIV testing and counseling during weekends. Health facilities to send Short Message Service (SMS) alerts, write formal invitation letters to invite male partners to the clinic for PMTCT and to accommodate the working class male partners who come for HIV testing and counseling with their wives during weekends. Making health facilities more male friendly in terms of space and services offered is also crucial.

### Public health actions taken

Findings of this study were shared with 80 provincial health team (PHT) members for Midlands province and as a result recommendations were taken up. The draft formal invitation letter for male partners was shared with PMTCT program managers. PMTCT educational quarterly campaigns were scheduled by the health promotion department in collaboration with the National AIDS Council and other partners.
